# Molecular Mechanism of Body Color Change in the Ecological Seedling Breeding Model of *Apostichopus japonicus*

**DOI:** 10.3390/biology14070873

**Published:** 2025-07-17

**Authors:** Lingshu Han, Pengfei Hao, Haoran Xiao, Weiyan Li, Yichen Fan, Wanrong Tian, Ye Tian, Luo Wang, Yaqing Chang, Jun Ding

**Affiliations:** 1 Liaoning Provincial Key Laboratory of Northern Aquatic Germplasm Resources and Genetics and Breeding, Dalian Ocean University, Dalian 116023, China; hanlingshu@dlou.edu.cn (L.H.); haopengfei0818@163.com (P.H.); xiaohr1218@163.com (H.X.); y99wy9@163.com (W.L.); fanyichen20240918@163.com (Y.F.); tianwanrong0615@163.com (W.T.); tianye0401@163.com (Y.T.); wangluo@dlou.edu.cn (L.W.); 2 Key Laboratory of Mariculture and Stock Enhancement in North China’s Sea, Ministry of Agriculture and Rural Affairs, Dalian Ocean University, Dalian 116023, China; yqkeylab@hotmail.com; 3 Dalian Jinshiwan Laboratory, Dalian 116034, China

**Keywords:** body color, molecular mechanism, chromatic biomarkers, ecological breeding, sea cucumber

## Abstract

The mismatch between the rapid expansion of breeding scale and outdated techniques has hindered the development of the sea cucumber (*A. japonicus*) industry. Our previous work revealed that ecological seedling breeding can produce red-colored *A. japonicus*, a phenotype not observed in traditional artificial breeding, where individuals are typically green. To investigate the molecular and genetic basis of this novel red coloration, we compared the growth conditions of red sea cucumbers and green sea cucumbers, as well as the differences in the pigment composition, gene expression and metabolites of their body walls. Red individuals showed higher body length and weight, and elevated levels of astaxanthin, lutein, canthaxanthin, and β-carotene in the body wall. Transcriptomic and metabolomic analyses identified differentially expressed genes and metabolites associated with pigmentation. In particular, *FMO2* and *WDR18*, involved in the cytochrome P450 drug metabolism pathway, were significantly upregulated in red individuals and are known to play roles in pigment biosynthesis and light signal perception. Key metabolites such as astaxanthin and fucoxanthin were implicated in body color formation. Moreover, genes in the arachidonic acid metabolism pathway were highly expressed, suggesting that dietary factors may contribute to pigment synthesis and accumulation. These findings provide novel insights into the mechanisms underlying body color variation in *A. japonicus* and offer potential for improved breeding strategies.

## Simple Summary

This study deciphers red coloration mechanisms in *Apostichopus japonicus* through multi-omics analysis. Red morphs exhibited enhanced growth and carotenoid accumulation (astaxanthin, lutein). Transcriptomics revealed upregulated flavin-containing monooxygenase 2 (*FMO2*) and WD repeat-containing protein 18 (*WDR18*) in cytochrome P450 pathways, controlling pigment biosynthesis and phototransduction. Metabolomics identified astaxanthin and fucoxanthin as key chromatic agents. Coordinated arachidonic acid metabolism activation suggests dietary precursors drive pigmentation. Findings of the study revealed that the red coloration in red sea cucumbers obtained via the ecological seedling model results from the synergistic interplay between gene expression regulation (such as CYP450 pathway-related genes *FMO2*/*WDR18*) and accumulation of key metabolites (e.g., astaxanthin). This research lays a theoretical foundation for understanding the mechanism of body color formation in sea cucumbers and provides insights into the molecular mechanisms underlying environmental adaptation in echinoderms.

## 1. Introduction

*Apostichopus japonicus is* an important economic species of marine culture in northern China, and market demand has been rising [1,2]. The existing production has been unable to meet the market demand, despite the annual output of sea cucumber being 248,508 tons in China in 2023 [3]. In this context, improving the breeding efficiency has become the core of guaranteeing the sustainable supply of sea cucumbers. Consequently, the research and development of ecological seedling breeding technology represents a key strategic initiative to promote the transformation of the aquaculture industry to ecological intensification and greening [4].

**Figure 1:**
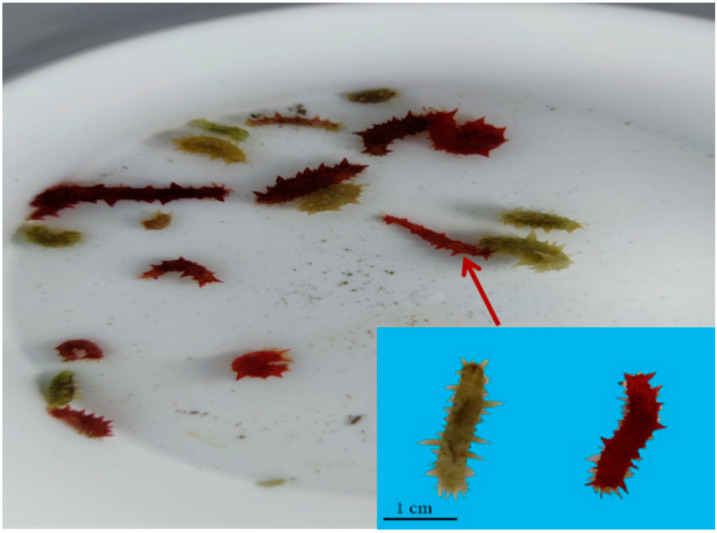
Reddening of body color under the ecological fry breeding mode of *Apostichopus japonicus*.

During research, exploring the ecological seedling breeding technology of *A. japonicus*, we found that some individuals turned red during the development stage to juvenile sea cucumber (Figure 1). Compared with the traditional completely dark culture conditions, exposure to light intensity was 2000–3000 Lx, which resulted in red juvenile sea cucumbers, with a color change rate reaching 95%. The preliminary observations revealed a growth advantage in the red morph compared to the green morph.

In sea cucumber aquaculture, body color variation has long been recognized as an important trait, with pigmentation being a key characteristic affecting growth and development [5]. The diverse colors exhibited by sea cucumbers not only reflect their adaptability to varied habitats but may also represent the external manifestation of distinct subspecies formed through long-term evolutionary isolation [6,7]. In natural environments, the red morph of *A. japonicus* predominantly inhabits open sea reef areas and intertidal algal fields, while the green morph is mainly distributed in inner bay waters [8]. Red, green, and black variants have been documented in Japan and Korea [9,10]. In China, however, *A. japonicus* populations remain predominantly green, despite the successful cultivation of white and purple strains [11]. Crucially, the phenomenon of individuals turning red had not been reported prior to this study. The novel findings presented herein fill a significant research gap in this field within China.

Based on these findings, this study aims to elucidate the molecular mechanisms underlying the reddening of body color in *A. japonicus* under an ecological seedling breeding model. We will employ liquid chromatography to precisely quantify the nutritional components within the body walls of red and green sea cucumbers. Utilizing RNA-seq technology, we will systematically detect and compare differences in growth and gene expression between the two color morphs, further identifying key metabolites associated with the reddening process. This research will not only enrich the theoretical understanding of body color in *A. japonicus* but also hold significant potential for providing crucial information to breed novel strains with superior growth performance. These advances are expected to help overcome key bottlenecks in the current sea cucumber farming industry and promote its sustainable development.

## 2. Materials and Methods

### 2.1. Experimental Materials

The material used in the experiment was 2-month-old *A. japonicus*, and its parent seed *A. japonicus* (weight 286.5 ± 0.36 g) was collected from Dalian Xinyulong Marine Biotechnology Co., Ltd., Dalian, China (39°20″ N, 122°19″ E) on 18 June 2022. After being stimulated by drying in the shade and flowing water, the sperm and eggs were discharged and fertilized for 40 min. The uplifted high-quality fertilized eggs were transferred to the hatchery with 300-mesh sieve gauze, and when the fertilized eggs developed into early-stage auricularia with a body length of about 450 ± 10 μm, they were taken out to carry out ecological seedling culture.

Establishment of an ecological seedling breeding model for sea cucumbers: The experimental water volume was 100 L. An adjustable aquatic plant light (Model: GM95-360280-D, Ningbo Moredian Biological Technology Co., Ltd., Ningbo, China) emitting full white light was suspended above the culture tank, with a photoperiod of 12 h light: 12 h dark (12 L:12 D). Light intensity was measured using a VICTOR 1010A illuminometer (Shengli Co., Ltd., Shenzhen, China). Four experimental light intensity groups were established: 1000 Lx, 2000 Lx, 3000 Lx, and 5000 Lx. Different treatment groups were separated using black plastic sheets. Initial larval stocking density was 0.2–0.5 individuals/mL. Based on the optimal growth environment for *A. japonicus* planktonic larvae (temperature 21.0–22.5 °C, pH 7.5–8.0, salinity 28–31‰, and dissolved oxygen 7–8 mg/L), water was not changed throughout the experiment in any group. Each experimental group received an addition of 500 mL of primary culture of the diatom *Chaetoceros muelleri* at a density of 1.5 × 10^6^ cells/mL. Under these conditions, *C. muelleri* was also able to grow and propagate. In this model, we found that when the light intensity was 2000–3000 Lx, compared with the traditional completely dark culture condition, the body color of juvenile sea cucumber was reddened, and the color change rate was up to 95%, and the red sea cucumber (1.66 ± 0.15 cm, 0.156 ± 0.5 g) might have a growth advantage over the green sea cucumber (1.56 ± 0.15 cm, 0.166 ± 0.5 g).

The sea cucumber samples used in this study were marine invertebrates and were handled in accordance with international ethical guidelines for invertebrate research without institutional ethical approval.

### 2.2. Measurement of Body Length and Weight of Different Body Colors of Sea Cucumber

To confirm the growth advantages of red sea cucumbers, 2-month-old sea cucumbers cultivated in this experiment had an average body length and weight of 1.55 ± 0.11 cm and 0.176 ± 0.05 g, respectively. In the experiments, the experimental group of red sea cucumbers with 3000 Lx of light (with a photoperiod of 12 L:12 D) (R) and the control group of green sea cucumbers with 0 Lx (W) were set up, and the continuous aeration culture method was used, and the bottom suction operation was performed once a day. The rearing period lasted 45 days. Feed for juvenile sea cucumber was special feed with sea mud feed. During the experiment, 10 individuals of red and green sea cucumbers were randomly sampled every 15 days.

### 2.3. Determination of Astaxanthin, Keratoxanthin, Lutein and Β-Carotene in Different Body Colors of Stichopus japonicum

The body wall of the sea cucumber was weighed and placed in a centrifuge tube, and 10 mL of methylene chloride:methanol (75:25) solution was added as the extraction solvent, and ultrasonic extraction was carried out for 20 min. After centrifugation at 8000/min for 5 min, the supernatant was taken, and the residue was repeatedly extracted with a solution of methylene chloride:methanol (75:25) until colorless. The combined extract was filled to 20 mL, filtered through a 0.45 μm microporous filter membrane, and detected by liquid chromatography. In the determination process, SHISEIDO C30 (4.6 mm × 250 mm × 5 μm) column and DAD detector were used, the column temperature was set at 30 °C, the injection volume was 10 μm, the wavelength was 450 nm, the flow rate was 1.0 mL/min, and the mobile phase was A: methanol:acetonitrile:water = 73.5:24.5:2, B: methyl tertbutyl ether [12]. The mobile phase gradient is shown in Supplementary Materials Table S1.

### 2.4. RNA Extraction, Library Preparation and Illumina Sequencing

RNA was extracted from 6 body wall samples of two species of sea cucumber using Trizol and Mirvana™ miRNA Isolation Kit, Ambion (USA), and the RNA quality was examined by 1.2% agargel electrophoresis to determine the possible degradation and contamination status. To ensure the purity of the RNA, the nano photometer spectrometer was used to monitor its purity, and the Qubit 2.0 fluorometer and Bioanalyzer 2100 system were used to detect RNA integrity and concentration [13]. The RNA was processed and crushed with NEBNext first strand synthesis reaction buffer, and mRNA fragments were used for cDNA synthesis. Six mRNA libraries of sea cucumber (two body colors R and W × three replicates) were constructed. QC was performed using the Agilent 2100 Bioanalyzer and ABI Step One Plus real-time PCR system, and sequencing was performed on Illumina Novaseq™ 6000 with a read length of two-ended 2 × 150 bp (PE150). All RNA clean data were submitted to the Short Read Archive (SRA) Sequence Database at the National Center for Biotechnology Information (NCBI) (Accession No. PRJNA1058950).

### 2.5. Quality Control and Assembly of Transcriptome Data

High-quality data were obtained by multiprocessing the raw data. First, the data were cleaned by removing adapters or reads containing more than 5% unknown bases (N) and low-quality reads. Next, after the screening of high-quality data for the Q20, Q30, GC content and sequence copy level of calculation, we obtain a reliable and accurate sequence of high quality. The Trinity algorithm combined these premium sequences to produce distinct transcript segments or unigenes. Redundancy from high-quality reads was eliminated to maximize data accuracy and efficiency [14]. We received more high-quality reads than the trepang genome-wide (Genme ID: 307972), and the the reference genome downloaded from (https://www.ncbi.nlm.nih.gov/datasets/genome/GCA_002754855.1/) (accessed on 6 November 2017).

### 2.6. Gene Function Annotation

The unigenes were annotated with the BLAST program (accessed via the NCBI BLAST web interface) using Swiss Prot, NCBI Nr protein and Nt nucleotide sequence databases, along with BLASTx and classified cell components, molecular functions, and biological processes using the NCBI Nr database in the Blast2GO program P [15]. InterPro Scan5 was used to obtain InterPro annotations. Finally, the unigene information was analyzed using the Gene Ontology (GO) and Kyoto Encyclopedia of Genes and Genomes (KEGG) approach [16].

### 2.7. Identification of Differentially Expressed Genes, Enrichment Analysis and qRT-PCR Validation

The pathway significance enrichment analysis bioinformatics method was used to select gene sets related to the KEGG pathway from the differential expression gene sets, and a hypergeometric test was used to compare the proportion of the two gene sets to find the significantly enriched pathway. The differentially expressed gene set was compared with the whole transcriptome background. *T*-test correction was used to calculate the significantly enriched pathway, and a *q*-value ≤ 0.05 was selected as the significant enrichment in differentially expressed genes.

To verify the correctness of the mRNA library, 10 differentially expressed genes (DEGs) were randomly selected from the mRNA library for verification. Roche LightCycler96 was used to perform qRT-PCR on randomly selected differential genes in the transcriptome library [17]. The primers used for quantification are shown in Table 1. The −2^−ΔΔCt^ method was used to calculate the relative expression of DEGs [18].

### 2.8. Extraction and LC-MS/MS Analysis of Body Wall Metabolites in Different Body Colors of A. japonicus

Body wall tissues of six red and six green sea cucumbers were collected for metabolite extraction. Each sample was approximately 100 mg. LC-MS/MS analysis using the LC-Q/TOF-MS platform (1290 Unlimited LC, 6530 UHD) and precision mass (Q-TOF/MS) liquid chromatography: The sample was separated by liquid chromatography to detect the target compound. The detailed processes of metabolite annotation and identification analysis were described in our previous study [19].

### 2.9. Data Processing and Statistical Analysis

CD3.1 software was used to process the raw data of the machine, and each metabolite was screened and quantified, the molecular formula was predicted and compared with the database, and finally, the identification and quantitative results of metabolites were obtained. We visualized using PCA and PLS-DA to obtain VIP values for each metabolite and then annotated these metabolites. Criteria with VIP value > 1, *p*-value < 0.05 and FC ≥ 2 or FC ≤ 0.5 were used to screen metabolites. Data processing is based on the Linux operating system and R, and Python software. In addition, MBRole (http://csbg.cnb.csic.es/mbrole/, accessed on 6 November 2017) was used to perform KEGG enrichment analysis of metabolites with significant changes. The calculated *p*-value is corrected by false discovery rate (FDR) to obtain the *q*-value. We took the *q*-value ≤ 0.05 as the threshold, and the pathway meeting this condition was defined as a significant enrichment pathway.

### 2.10. Combined RNA-Seq and Metabolome Analysis

From RNA-seq and LC-MS/MS results, the DEGs and modified metabolites were used to identify the biological pathway by using KEGG and GO, and data were converted into vectors to calculate Pearson’s correlation coefficient between metabolite–metabolite, gene–gene, and metabolite–gene profiles [20].

### 2.11. Statistical Analysis

The Levene test was used to analyze the homogeneity of variance. The results are expressed as the mean ± SEM. After confirming normality by Shapiro–Wilk tests, significant differences (*p* < 0.05) for each variable were first detected using the one-way ANOVA test, followed by Tukey’s HSD. The data was analyzed by SPSS 25.0. The figures were drawn using Origin 9.1 software.

## 3. Results

### 3.1. Differences in Growth and the Pigment Content of the Body Wall of Red and Green A. japonicus

After 30 days of breeding, differences in body weight and body length of red and green sea cucumbers were detected (Table 2). The body length and body weight of the red sea cucumber were significantly higher than those of the green sea cucumber (*p* < 0.05).

To explore the red origin of *A. japonicus*, the astaxanthin, lutein, canthaxanthin, and β-carotene content in the body wall of the sea cucumbers was obtained by liquid chromatography. The results showed that the contents of the four pigments in the body wall of red sea cucumber were significantly higher than those of green sea cucumber (Table 3), and the contents of β-carotene and astaxanthin were significantly different (*p* < 0.01).

### 3.2. Analysis and Functional Annotation of Differentially Expressed Genes (DEGs)

Transcriptome sequencing (RNA-Seq) of the body walls of red *A. japonicus* (R1, R2, and R3 experimental groups) and green *A. japonicus* (W1, W2, and W3 control groups) was performed. A total of 258,171,718 raw reads were obtained from the six samples. After filtering the raw sequencing data, 253,656,928 valid clean reads were obtained. The proportion of Q20 bases was 97.22–97.60%, the proportion of Q30 bases was 92.45–93.44%, and the GC content was 38.34–40.85%. The clean reads were mapped to the reference genome with comparison rates of 67.33–69.89% and 66.06–68.12%, respectively (Supplementary Table S2).

**Figure 2:**
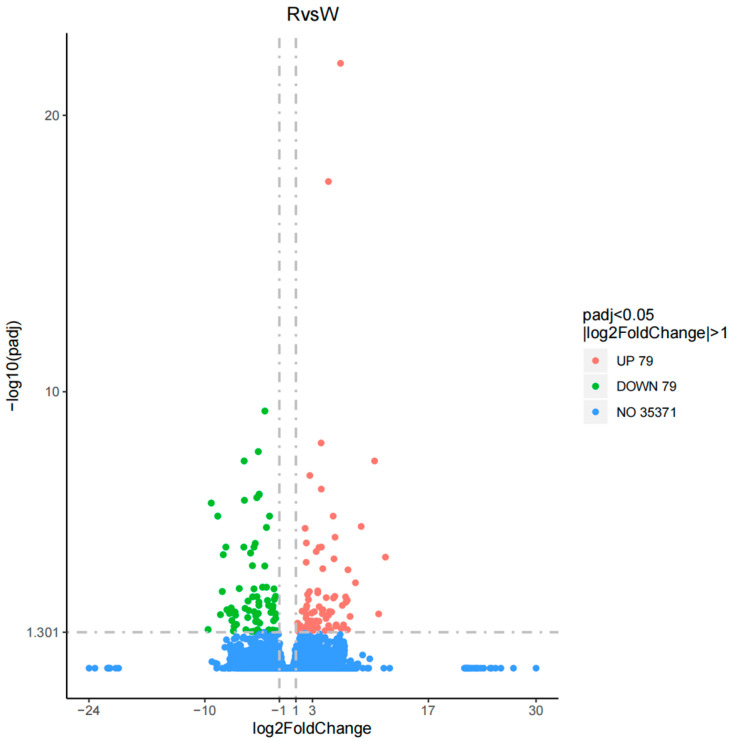
Volcano map of differential expression analysis of the body wall of red and green *A. japonicus*.

The DEGseq software package was used to analyze the differential expression of genes in the body wall of red and green *A. japonicus* with |log_2_(fold change)| ≥ 1 and *p* ≤ 005. A total of 158 DEGs were detected in the experimental versus control (R vs. W) comparisons; 79 DEGs were upregulated and 79 DEGs were downregulated (Figure 2).

**Figure 3:**
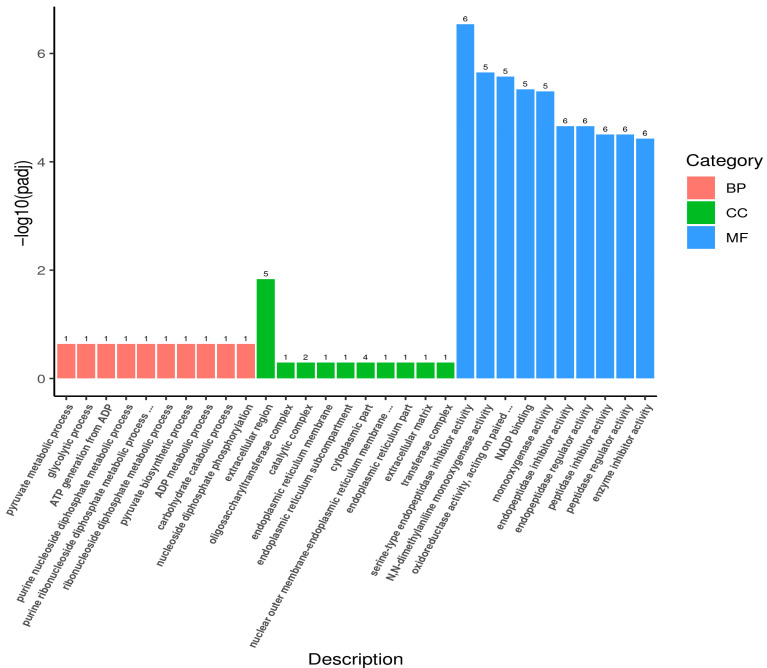
Bar plot of GO enrichment analysis for differentially expressed genes in the body wall of body wall of red and green *A. japonicus*.

**Figure 4:**
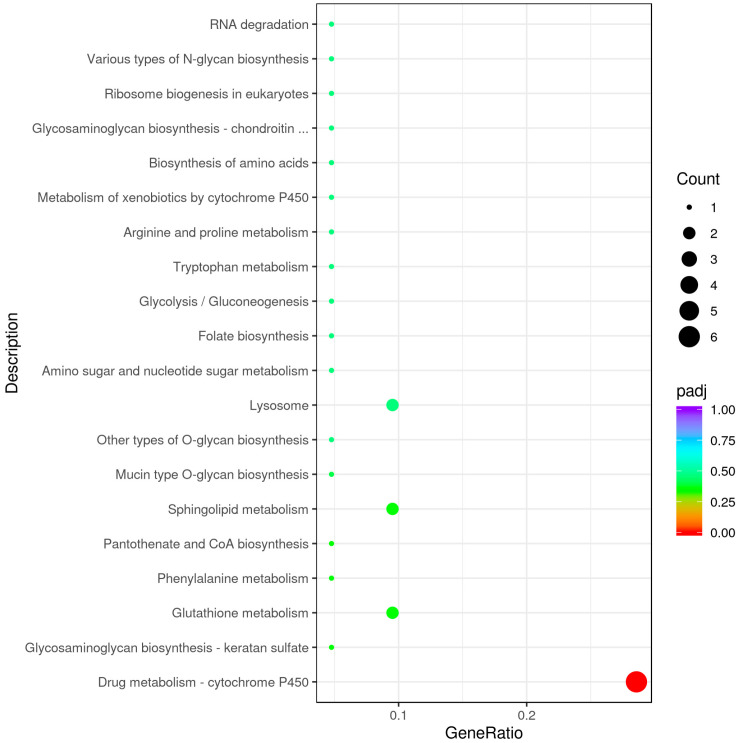
Scatter plot of enrichment of differential gene KEGG pathway in the body wall of red and green *A. japonicus*.

The GO functional enrichment analysis of the DEGs showed that DEGs were annotated with 177 GO terms that were classified into 30 subcategories under the 3 main GO categories of biological process (BP), cellular component (CC), and molecular function (MF) (Figure 3). In the R vs. W comparison, pyruvate metabolic process, glycolytic process, and purine nucleoside diphosphate metabolic process were significantly enriched under BP (*p* < 0.05); the extracellular region was significantly enriched under CC (*p* < 0.05); and serine-type endopeptidase inhibitor activity, NADP binding, and N-dimethylaniline monooxygenase activity were significantly enriched under MF (*p* < 0.05). The KEGG pathway enrichment analysis of the DEGs showed that 34 DEGs were associated with 26 pathways, with drug metabolism-cytochrome P450 (5 upregulated and 1 downregulated DEGs) being the most significantly enriched (Figure 4).

### 3.3. qRT-PCR Validation of DEGs

**Figure 5:**
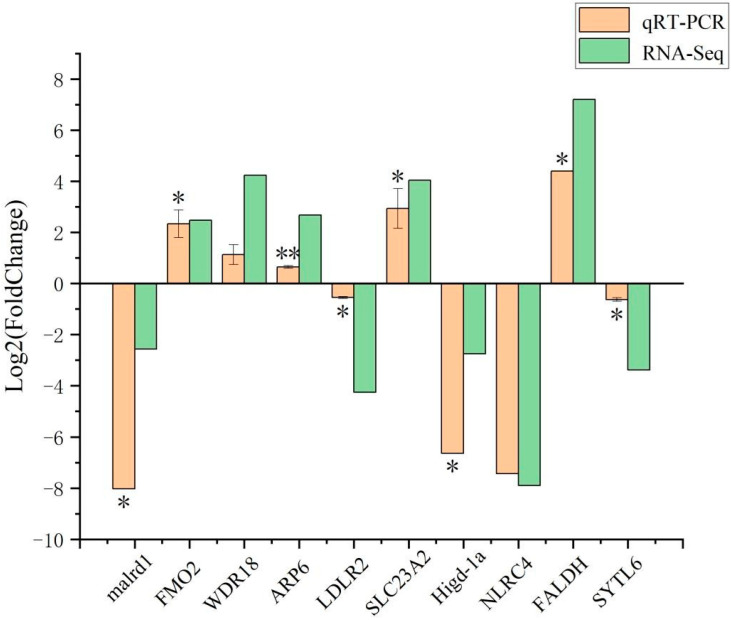
qRT-PCR verification results and the values are expressed as the mean standard deviation (*n* = 3), * indicates significant difference between R and W (*p* < 0.05), ** indicates extremely significant difference between R and W *(p* < 0.01).

Ten DEGs were selected randomly to verify the transcriptome sequencing results. The expression changes obtained by qRT-PCR were compared with the transcriptional profiles obtained by RNA-seq (Figure 5). The trends in expression obtained by RNA-seq and qRT-PCR analysis were consistent for all 10 genes, which confirmed the reliability and accuracy of the RNA-seq data.

### 3.4. Principal Component Analysis and Pls-Da of Body Wall Metabolic Profiles of Red and Green A. japonicus

**Figure 6:**
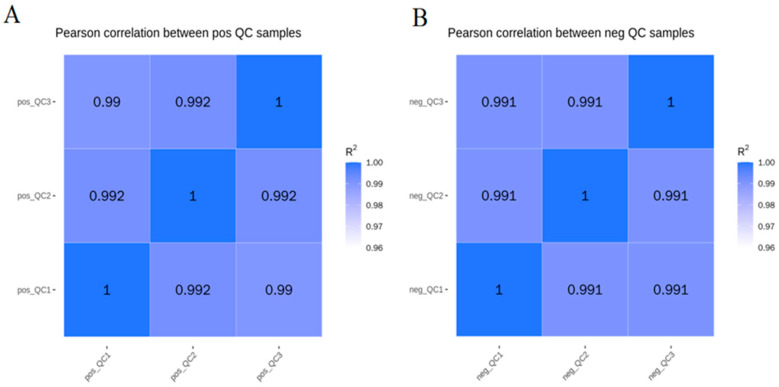
Pearson correlation of QC samples, (**A**) is positive ion mode, (**B**) is negative ion mode, and R2 is the coefficient of determination.

**Figure 7:**
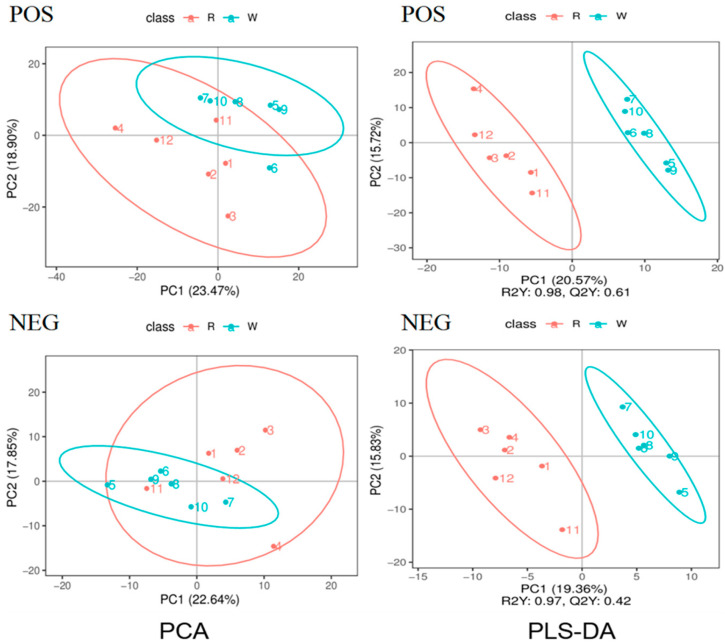
PCA and PLS-DA analysis of the body wall of red and green *A. japonicus*, POS stands for positive ion mode (**up**), and NEG stands for negative ion mode (**down**).

The differential metabolic profiles of the body walls of red and green sea cucumbers were obtained by liquid chromatography with tandem mass spectrometry (LC-MS/MS). In the LC-MS/MS analysis, the Pearson correlation coefficient of the QC samples was 0.99–0.992 in the positive (POS) model and 0.991 in the negation (NEG) model, indicating high data stability and quality (Figure 6). The principal component analysis showed there were no outliers in the score plot for each sample, indicating high sample quality (Figure 7). A partial least squares-discriminant analysis (PLS-DA) of the red and green sea cucumber samples was also conducted. The *R2* (model interpretation rate) values of R and W in the POS model were 0.98, Q2 (used to evaluate the prediction ability of the PLS-DA) was 0.61; and the *R2* values of R and W in the NEG model were 0.97, Q2 value was 0.42. All the *R2* values were higher than the Q2 values. Overfitting tests showed that the model was reliable, indicating that the model can be used for subsequent analysis (Supplementary Figure S1).

### 3.5. Identification of Metabolites in the Body Walls of Red and Green A. japonicus

In this study, non-targeted metabolomics techniques were used to obtain metabolome data of the body walls of red and green *A. japonicus*. A total of 579 positive ion-mode metabolites and 253 negative ion-mode metabolites were identified from 12 samples. Functional identification and annotation of the metabolites were conducted using the KEGG database and the Human Metabolome Database (HMDB). Environmental information processing was enriched in the first-level KEGG analysis. All positive ions were enriched in signaling molecules and interactions and membrane transport, and all negative ions were enriched in membrane transport in the second-level analysis. Genetic information processing was also enriched in the first-level analysis; all positive and negative ions were enriched in translation in the second-level analysis. Under the first-level classification of metabolism, all the pathways that were enriched in the second-level analysis for all the positive and negative ions are shown in the global and overview maps (Supplementary Figure S2). In the HMDB analysis, lipid and lipid-like molecules were the most enriched metabolites in the positive ion mode, with a total of 87 metabolites annotated, followed by organic acids and derivatives and organoheterocyclic compounds with 57 and 42 metabolites, respectively. In the negative ion mode, lipid and lipid-like molecules were also the most enriched metabolites in the negative ion mode with a total of 57 metabolites annotated, followed by nucleosides, nucleotides, and analogues, and organoheterocyclic compounds with 15 and 10 metabolites, respectively. These results show that, in the positive and negative ion modes, lipid and lipid-like molecules and organic heterocyclic compounds were the most enriched metabolites (Supplementary Figure S2).

**Figure 8:**
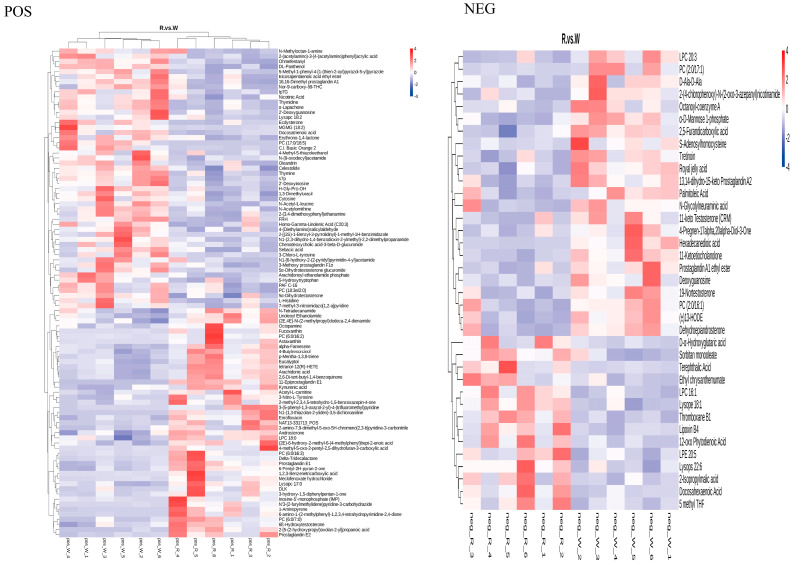
SDM clustering thermogram of the body wall of red and green *A. japonicus*, POS stands for positive ion mode (**left**) and NEG stands for negative ion mode (**right**).

**Figure 9:**
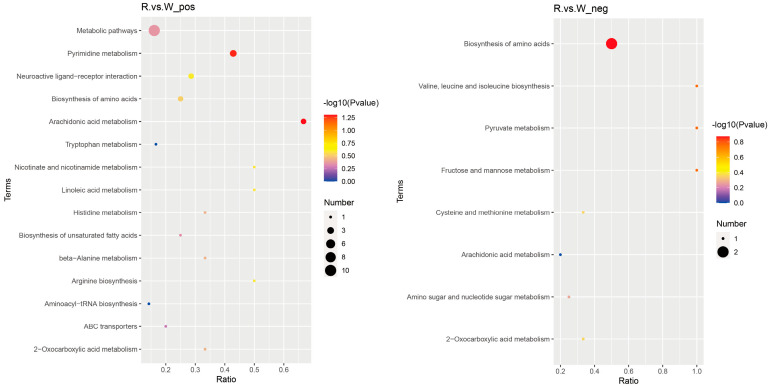
Bubble diagram of KEGG enrichment of different metabolites in the body wall of red and green *A. japonicus*, POS stands for positive ion mode (**left**) and NEG stands for negative ion mode (**right**).

To reduce the number of candidate metabolites, we used a reference sequence comparison method. We used Bismark software to map bisulfite-treated clean reads to the reference genome and perform methylation calls [21]. The C-site coverage under each context (CpG, CHG, CHH) was calculated. A total of 133 differentially abundant metabolites (SDMs) were selected; 96 were in positive ion mode and 37 were in negative ion mode, and 59 were increased and 74 were decreased. Hierarchical cluster analysis of the 133 candidate DAMs was performed to determine their roles in the body walls of red and green sea cucumbers (Figure 8). The KEGG enrichment analysis of the DAMs found that 29 DAMs in the positive ion mode were annotated in 15 pathways, the most significantly enriched being arachidonic acid metabolism. Nine DAMs in the negative ion mode were annotated in eight pathways, the most enriched being biosynthesis of amino acids (Figure 9).

### 3.6. Combined Transcriptome and Metabolome Analysis of the Body Walls of Red and Green A. japonicus

**Figure 10:**
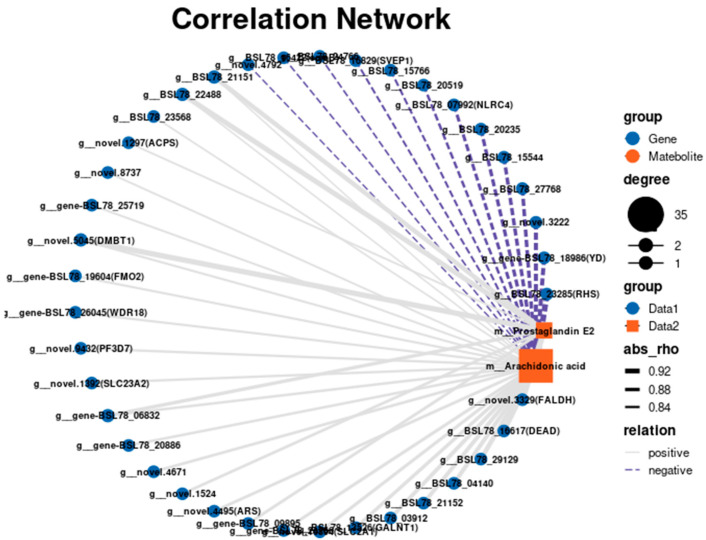
Correlation network diagram of differentially expressed genes and metabolites in the body wall of red and green *A. japonicus*. The circles are differentially expressed genes, the squares are differentially expressed metabolites, and the solid gray lines show that there is a positive correlation between them. The blue dotted line indicates that there is a negative correlation between them.

To further explore the association between metabolites and genes in the R vs. W comparison, we performed a joint transcriptomic and metabolomic analysis. We found that arachidonic acid metabolism was a significantly enriched KEGG pathway. The interaction network diagram between two metabolites and genes in the arachidonic acid metabolic pathway was visualized based on the analysis results (Figure 10). A correlation coefficient *p*-value < 0.05 was considered to be significant. A Pearson coefficient < 0 is considered a negative correlation and >0 is considered a positive correlation. The differentially abundant metabolite arachidonic acid was associated with 35 key DEGs; 22 were positively correlated and 13 were negatively correlated. The differentially abundant metabolite prostaglandin E2 was associated with eight key DEGs, all of which were positively correlated.

## 4. Discussion

The comparison of the body lengths and body weights of red and green *A. japonicus* after 30 days of cultivation showed that the body length and body weight of red sea cucumber were significantly higher than those of green sea cucumber, which are important quality characteristics of red *A. japonicus*. The liquid chromatography results showed that the content of four pigments in the body wall of red *A. japonicus* was significantly higher than that of green *A. japonicus* (*p* < 0.05), among which the β-carotene and astaxanthin content was significantly different (*p* < 0.01). Astaxanthin is a potent colorant and antioxidant, and its powerful antioxidant activity suggests its potential to treat a variety of health conditions [22]. β-carotene is an important source of vitamin A, and it also has antioxidant properties and immune-enhancing effects [23]. Adding astaxanthin-rich yeast, algae, or carotenoids to aquatic animal feed has been shown to effectively improve body color [24]. By comparing the differences in astaxanthin abundance between common *A. japonicus* and albino *A. japonicus* at different developmental stages, the abundance of astaxanthin in the body wall of albino *A. japonicus* was found to be significantly lower than that of common sea cucumber, implying that astaxanthin abundance was closely related to color change in *A. japonicus* [25]. We also showed that the astaxanthin content in the body wall of red *A. japonicus* was significantly higher than that of green *A. japonicus*, indicating that the red characteristic of *A. japonicus* was related to a high abundance of astaxanthin.

We constructed and sequenced transcriptome libraries of red and green *A. japonicus* to detect and compare dynamic changes in mRNA expression in the body walls of red and green *A. japonicus*. The KEGG pathway enrichment analysis of the DEGs showed that they were significantly enriched in the drug metabolism-cytochrome P450 pathway. Cytochrome P450 (CYP450) is a heme protein that is widely distributed in vertebrates, invertebrates, and plants. It plays an important role in the metabolism of endogenous substances, drugs, and exogenous substances, and has an important impact on cytokines and body temperature regulation [16,26,27]. This study found that the *FMO2* and *WDR18* in the CYP450 pathway were upregulated. Dimethylaniline monooxygenase [N-oxide-forming] 2 (FMO2) is a mitochondria-specific factor involved in pigment cell expression but not in pigment biosynthesis [28]. FMOs play a crucial catalytic role in the oxidation of pigment precursors in echinoderms. Calestani et al. [29] isolated specific genes, including PSK and FMO, from embryos of the purple sea urchin (*Strongylocentrotus purpuratus*), which are expressed exclusively in pigment cells. Knockdown of the *Sp-Fmo* gene resulted in a white differentiation phenotype in some sea urchin larvae. This confirmed the involvement of the *FMO* gene in pigment biosynthesis in sea urchins. A study found that purple sea cucumbers and green sea cucumbers lighten their own color under light conditions. By analyzing the transcriptional expression of light-colored spiny sea cucumber genes in normal spiny sea cucumbers, it was found that the expression of the *FMO2* gene was upregulated in the light purple sea cucumbers, suggesting that the *FMO2* gene may lead to the pigmentation of sea cucumbers. This result is consistent with the findings of study [30]. *WDR18* is a member of the *WD40* gene family. The WD40 protein is highly conserved and can be involved in various biological mechanisms, such as cell division, programmed cell death, light signal transduction and perception, etc. [31]. In plants, WD40 has been reported to inhibit light signal transduction and affect pigment synthesis [32]. However, WD has not been reported in the study of echinoderm pigments. In this study, the expression level of the *WD*R18 gene in red sea cucumbers cultivated under light was upregulated. It is speculated that the *WD*R18 gene plays an important role in the response of sea cucumbers to light signals and changes in body color. These research results indicate that the upregulation of FMO2 and WDR18 may have affected the color changes of sea cucumbers. These findings are more conducive to the light conditions and breeding mode required by the body color of the *A. japonicus*, and are applied to the selection of high-quality varieties.

Metabolomics can be used to analyze the change rules, interactions, and relationships among physiological and pathological processes based on the overall and dynamic changes of all metabolites in the body [33]; therefore, we used metabolomics to study the changes in the body color of *A. japonicus*. Among the 133 DAMs identified in this study, the abundance of 59 DAMs increased and the abundance of 74 DAMs decreased in red sea cucumber compared with their abundance in green sea cucumber. Most of the DAMs identified were lipid and lipid-like molecules, including fucoxanthin and astaxanthin, which showed increased abundance. Astaxanthin is a non-vitamin A carotenoid that is a very dynamic pigment that can accelerate the growth rate of aquatic animals and improve their survival rate, and is a strong antioxidant with a bright color [34]. Many aquatic animals, including coelenterates, crustaceans, mollusks, echinoderms, and fish, contain astaxanthin-rich components [35,36,37,38,39]. However, aquatic animals cannot synthesize astaxanthin, and mainly accumulate astaxanthin in their bodies by feeding on algae or other microorganisms that contain astaxanthin [40]. Astaxanthin is stored temporarily as astaxanthin esters or carotenoid protein complexes after entering the tissues of animals [41]. Astaxanthin plays an important role in maintaining the vivid color of aquatic organisms. By adding synthetic astaxanthin to the feed, the study found that the muscle redness of *Pterophyllum scarale* significantly increased [27]. Mora et al. [42] found that 6 weeks after rainbow trout consumed a diet containing 80 mg/kg astaxanthin, muscle redness and yellowness were significantly increased. During the sexual maturation of the *Panulirus cygnus*, the content of astaxanthin metabolites (in the form of astaxanthin esters) in the red shell of this lobster was 2.4 times that in the white shell of the newborn [43]. In addition, unlysed carotenoids in the animal gut were decomposed into oxidized polar products by the CYP450 family enzyme system and excreted [44], which was consistent with the transcriptomic results.

Fucoxanthin is a carotenoid with a unique structure and is found mainly in large brown marine algae (such as Laminaria and *Undaria pinnatifida*) and marine microalgae (such as *Chaetoceros muelleri* and *Phaeodactylum tricornutum*) [45]. In aquatic animals, fucoxanthin compounds have important functions, such as acting as a colorant for the body and tissues, and enhancing the adaptability of some organisms to the environment [46]. Fucoxanthin and its metabolites can also induce the apoptosis of human PC-3 prostate cancer cells by the activation of cysteine proteinase-3 [26]. Although aquatic animals cannot synthesize carotenoids from scratch, they can obtain them by modifying carotenoids in their food. Bivalve shellfish obtain carotenoids from food microalgae and modify them through metabolic reactions, and their body contains a variety of carotenoids with diverse structures [47]. Symonds found a large amount of fucoxanol in the intestinal wall of *Psammechinus miliaris*, indicating that fucoxanthin, the main carotenoid in brown algae, was degraded in the intestinal tract of these sea urchins, implying their main pigment came from carotene in brown algae [48]. In the present study, fucoxanthin abundance was significantly increased in red *A. japonicus*, suggesting that it plays a key role in the change of body color. In the early stage of the study, the ecological seedling breeding model of *A. japonicus* was established based on sunlight, microalgae, and *A. japonicus*. The open feed of *A. japonicus*, C. muelleri, is rich in fucoxanthin. Therefore, we speculated that under light, C. muelleri grew faster, the food intake of *A. japonicus* increased, the growth metabolism accelerated, pigment accumulation was promoted, and the body color changed to red. This implies that the food source is one of the causes of the red color of *A. japonicus*.

The combined transcriptome and metabolome analysis showed that the abundance of arachidonic acid (ARA) was significantly expressed in the ARA metabolic pathway. ARA is a highly unsaturated fatty acid that belongs to the N-6 series. Approximately 40% of the total unsaturated fatty acids in aquatic animals have been shown to be ARA, indicating that this nutrient plays an irreplaceable role in the infancy of animals [49]. Studies have shown that ARA plays an important role in the growth, stress resistance, immune and reproductive performance, and pigmentation of aquatic animals. For example, increased ARA levels have been shown to significantly improve the pigmentation status of fish [50]. A study of the effects of eicosapentaenoic acid (EPA) and ARA on pigmentation in juveniles and larvae of *Hippoglossus hipoglossus L.* and *Scophthalmus maximus L.* showed that ARA rather than EPA was the main factor affecting pigmentation. However, high ARA levels have negative effects on the pigmentation of fish; therefore, it is particularly important to provide an appropriate ARA diet to maintain normal formation and pigmentation of the back skin when feeding juvenile flounder (*Paralichthys olivaceus*) in the open [51]. ARA is a precursor of the active substance prostaglandin E2 (PGE2). Lund found that ARA-derived PGE2 significantly affected the body color of *Solea sole L.*, whereas environmental factors such as light intensity and tank color had little effect [52]. We found that ARA was positively correlated with the expression of *FMO2* and *WDR18*, which were found to be involved in pigment synthesis in *A. japonicus*. Therefore, we speculated that ARA is closely related to PGE2 and the red body color of *A. japonicus*. Further studies are needed to confirm these findings.

## 5. Conclusions

In a previous study, we found that the body color of *A. japonicus* changed to red under an ecological seedling breeding model. To understand the cause and molecular mechanisms involved in the formation of red body color, we measured changes in body weight, body length, and pigment content between red and green *A. japonicus,* and constructed body wall gene expression and metabolite profiles of red and green *A. japonicus*. The cytochrome P450 pathway, in which *FMO2* and *WDR18* participate in pigment biosynthesis and light signal transmission and perception, was found to play an important role in the process of body color change. Key metabolites, including astaxanthin and fucoxanthin, were found to play key roles as body and tissue colorants in the body color change of *A. japonicus*. The combined transcription level and metabolic level analysis showed that the metabolic pathway of ARA was significantly expressed, implying that the food source was important for the red body color change of *A. japonicus*. In the future, we will optimize the ecological seedling breeding model of *A. japonicus*, and further explore the molecular mechanism of body color change.

## Figures and Tables

**Table 1:** qRT-PCR analysis of primers for determining R vs. W comparative group genes.

Gene ID	Forward Primers (5′-3′)	Reverse Primers (3′-5′)
*malrd1*	TCACGAGACCAGGTACAGATGTTC	GAAGGAAGGAAACCATACGCTCAG
*FMO 2*	GACTGCCTCCCCGCTCTCC	TCGCAGCACGGTAGTATAACACATC
*WDR18*	ACATAGCGGAGCGTCAATAGGTAC	TGAAGGTTGAGTGAGGTCAAGTTCC
*ARP6*	TGTATAGCCTAAGCAACGAAGAGTC	CCGCACAGTACCTACAGTAATCAC
*LDLR2*	CGCACAGAATACAGACAGGTTAATG	TTATCTCCTACCGCTCCTCTTCC
*SLC23A2*	GCAGCGAATACTACGGAGGTTG	GTCCCGATGAGTCCAGTGAAAC
*Higd-1a*	CATGCTGGCACACTTGTATTGAG	CTATTACGACTACGAGGCTCTTGG
*NLRC4*	AGGGCGATTAAGTGTTTCTTCTCTC	GACGGACGAGCAACTACGAATG
*FALDH*	CTCCGCTCGCCATACATACATTC	CATTAAGACCCTCTCCCGCATAAAC
*SYTL6*	TTAGGCAAGTGGTGATGGTGAAC	GTGAGCAGACAACTGTGATGAATG
*CYTB*	TGACAGGACCGCTACGAAAGAGG	AAAGTTTTCTTGGGGCCGGAAGG

**Table 2:** Changes in body weight and body length of red and green *A. japonicus*.

Groups	Weight (g)			
	0 d	15 d	30 d	45 d
R	0.175 ± 0.03	0.247 ± 0.04	0.343 ± 0.05	0.78 ± 0.06 ^ab^
W	0.173 ± 0.02	0.201 ± 0.07	0.251 ± 0.06	0.56 ± 0.04 ^b^
Groups	body length (cm)			
R	1.550 ± 0.11	1.723 ± 0.14	2.524 ± 0.15 ^a^	3.152 ± 0.11 ^a^
W	1.521 ± 0.10	1.604 ± 0.13	1.963 ± 0.23 ^b^	2.848 ± 0.17 ^b^

Note: The data in the table are presented as mean ± standard deviation with *n* = 3. Different letters indicate significant differences among experimental groups (*p* < 0.05).

**Table 3:** Contents of four pigments in the body wall of red and green *A. japonicus*.

Serial Number	Sample Name	Astaxanthin (mg/kg)	Lutein(mg/kg)	Canaxanthin (mg/kg)	β-Carotene (mg/kg)
1	R	2.111 ^ab^	0.447 ^a^	0.554 ^a^	2.379 ^ab^
2	W	0.217 ^b^	0.057 ^b^	0.038 ^b^	0.066 ^b^

Note: The data in the table are presented as mean ± standard deviation with *n* = 3. Different letters indicate significant differences among experimental groups (*p* < 0.05).

## Data Availability

The original contributions presented in the study are included in the article; further inquiries can be directed to the corresponding authors.

## Supplementary Materials

The following supporting information can be downloaded at: https://www.mdpi.com/article/10.3390/biology14070873/s1, Figure S1: Fitting inspection diagram of *A. japonicus* body wall with different body colors. POS stands for positive ion mode (up), NEG stands for negative ion mode(down); Figure S2: Identification of different metabolites in body wall of a. japonicus with different body color; Table S1: Mobile Phase Gradient Table; Table S2: Statistics of the transcriptome sequencing results of the body wall of red and green *A. japonicus*.
