# Supplementary material for: Molecular Mechanism of Body Color Change in the Ecological Seedling Breeding Model of Apostichopus japonicus
Source: Biology (Basel). 2025 Jul 17;14(7):873. doi: 10.3390/biology14070873 (PMC12293039; doi:10.3390/biology14070873)
Supplement: Supplementary file 1 [file biology-14-00873-s001.zip › biology-3614784-supplementary.pdf]

Supplementary Materials

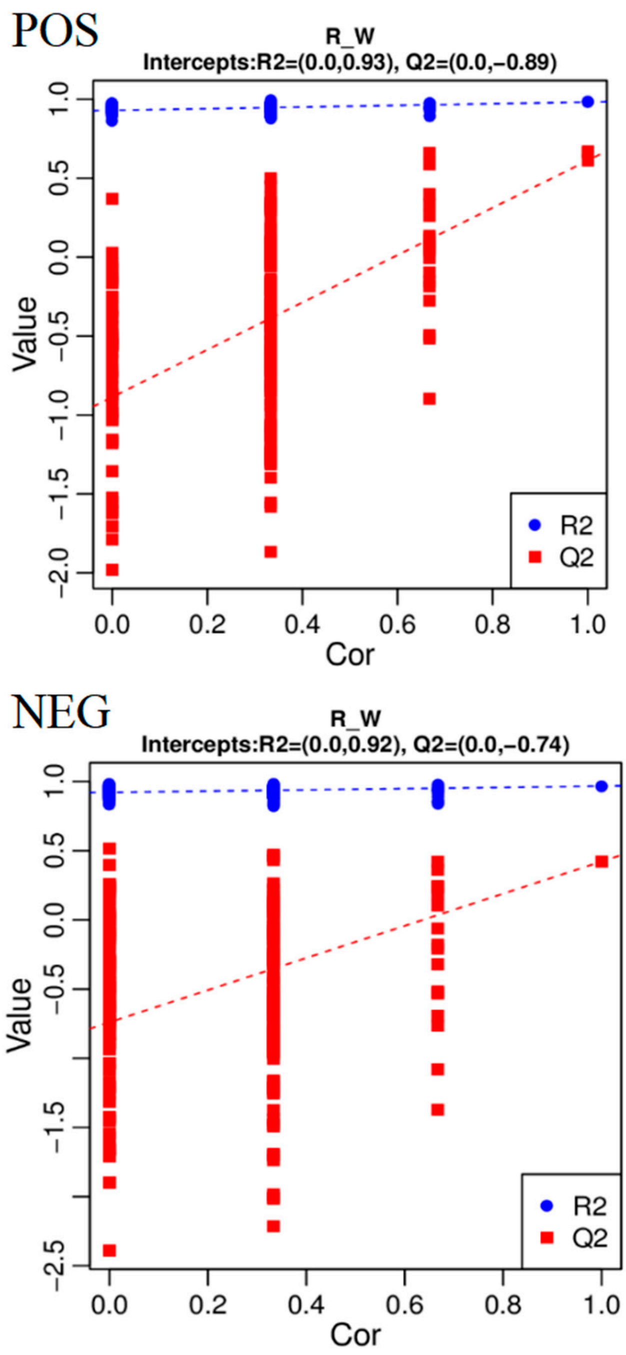

Figure S1: Fitting inspection diagram of *a. japonicus* body wall with different body colors. POS stands for positive ion mode (up), NEG stands for negative ion mode(down).

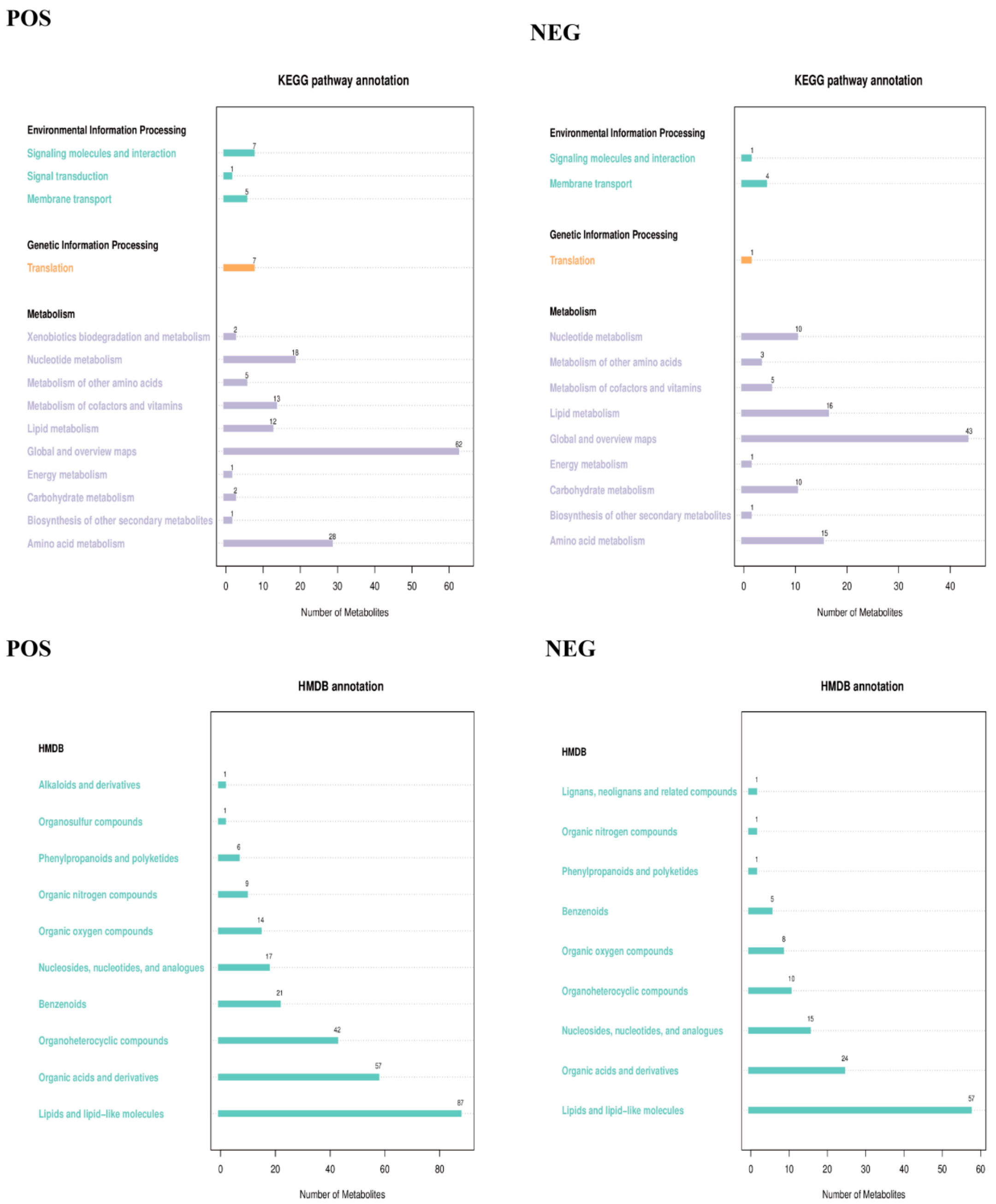

Figure S2: Identification of different metabolites in body wall of a. japonicus with different body color.

Table S1: Mobile Phase Gradient Table

Time (min)	Flow velocity (mL/min)	Mobile phase A (%)	Mobile phase B (%)
0.0	1.0	100	0
15.0	1.0	59	41
18.0	1.0	20	80

19.0	1.0	20	80
20.0	1.0	0	100
22.0	1.0	100	0
27.0	1.0	100	0

Astaxanthin, canthaxanthin, lutein, β-carotene content is calculated according to the formula: $W = \frac{(C - C_0) * V * N}{m}$

In the formula: W: sample target content, unit: mg/kg; C: Determine the concentration of the target substance in the liquid, unit: mg/L; C0: concentration of the target in the blank control, unit: mg/L; V: constant volume, unit: mL; N: dilution ratio; m: Sample quantity, unit: g.

Table S2: Statistics of the transcriptome sequencing results of the body wall of red and green *A. japonicus*

Sample	Raw Reads	Valid Reads	Valid Bases	Valid (%)	Q20 (%)	Q3 (%)	GC (%)
R1	39291652	38521936	5.78G	99.97	97.6	93.34	38.34
R2	43660856	42925594	6.44G	97.42	97.39	92.91	40.85
R3	44408412	43699806	6.55G	97.44	97.44	93.04	40.68
W1	44360000	43587400	6.54G	97.25	97.6	93.44	40.33
W2	44962612	44073928	6.61G	99.97	97.53	93.24	40.12
W3	41488186	40848264	6.13G	97.57	97.22	92.45	40.41
Total	258171718	253656928					
